# Reflections on a Name

**DOI:** 10.6004/jadpro.2016.7.7.1

**Published:** 2016-11-01

**Authors:** Heather M. Hylton

**Affiliations:** Ms. Hylton is a physician assistant at Memorial Sloan Kettering Cancer Center, New York, New York.

When autumn comes, I often find myself reflecting upon the rich and fascinating history of advanced practitioners, in particular, that of the PA. Perhaps it is because at the time of the writing of this piece, we have just concluded the annual celebration of National PA Week. Established in 1987, National PA Day, celebrated October 6 of every year, marks the graduation of the first class of physician assistants (PAs) from the Duke University PA Program (Physician Assistant History Society, 2016). We now honor the profession from October 6–12 each year with National PA Week, which was recognized with a Presidential Proclamation in 2010 (The White House, 2010).

An understanding of the nomenclature of our profession stems from an understanding of the forces driving the emergence of our profession 5 decades ago. Quite simply, the profession was named through the objective that was achieved by the concept of the profession itself—an assistant to a physician. Contextually, it was meant to refer to a skilled clinician who was competent to manage many aspects of the work typically done by the physician. Interestingly, the name of the profession was determined not by PAs themselves but rather by physicians, educators, advocates of the concept of the profession, and regulators (Hooker, Cawley, & Asprey, 2010). Even today, confusion over the name can be seen in clinical settings, as staff members in these environments are considered to be assistants to physicians but are not PAs. With the globalization of our profession, there is further confusion on nomenclature due to international differences in terminology to describe medical personnel in other countries.

## CONTROVERSY OVER AN OPTIMAL TITLE

From early on through the present day, the optimal title of the profession has been controversial. At the core of this issue is the philosophy that the title "Physician Assistant" is not considered to be an adequate or appropriate descriptor of the knowledge, expertise, and competency of the PA as well as the high level of responsibility inherent in the role. Over time, other names for the profession have been proposed. "Physician’s Assistant" was a title understandably taken out of commission given the implications of possession. The Yale School of Medicine has long embraced the title "Physician Associate," as evidenced by the name of its PA training program. The University of Washington School of Medicine’s PA training program, MEDEX, comes from the contraction of the term Medical Extender. Other titles proposed for the profession over time have included Syniatrist, Osler, and Flexner (Hooker et al., 2010).

So where does this leave us? The American Academy of PAs (AAPA) favors the use of the title "PA" over "Physician Assistant," as the latter is not thought to be in alignment with how PAs practice clinically and provide medical services to patients (AAPA, 2016). I have begun incorporating this nomenclature into both professional and social discussions, although sometimes with mixed results.

For example, earlier this year, I was socializing with a family of entrepreneurs with whom I had just become acquainted. During our conversation, they asked me what my profession is, to which I responded, "I’m a PA." Later that afternoon, they asked me, "What is it like being a Public Attorney?" As in many other cases, this turned into a wonderful opportunity to provide education about our profession. With increasing frequency, however, I find the public, while not necessarily familiar with the term "PA," is familiar with the Physician Assistant profession and commonly has received care from a PA, known a PA, or known someone who aspires to be a PA.

## EDUCATING THE PUBLIC AND OUR PATIENTS

Having been a PA for 15 years, I can certainly say the education I need to provide about my role in the health-care team is significantly less now than when I initially started in clinical practice. And how I speak about my role has changed as well—from practicing medicine under the supervision of a physician (language early on) to practicing medicine collaboratively with physicians and other health-care professionals as part of a team (language today). There remains an ongoing need, however, to educate the public about the educational preparation, licensure, and scope of practice of PAs and the critical role we serve as vital members of the patient’s care team.

I do not doubt the ideal title of our profession will continue to be debated. Implementation of a formal title change, however, would come with its challenges. In addition to requiring large-scale educational efforts to inform the general public about such a change, there would be the policy aspects of incorporating such changes as well. Not only would relevant laws and regulations need to be updated with the new language, the changes would need to be incorporated into facility policies, payer policies, and so forth. Incorporating a formal change in our professional name would be nothing short of a tremendous undertaking.

Shakespeare reminded us that a rose by any other name would smell as sweet—likewise, changing our name does not change what we as PAs do at the bedside. Continued efforts to educate our patients and the general public about our profession are needed, although I believe this is separate from the nomenclature issue. A major change in our professional title would come at a significant cost; the question that needs to be answered is, what value would such a change bring to our patients?
